# Comparisons of hearing threshold changes in male workers with unilateral conductive hearing loss exposed to workplace noise: a retrospective cohort study for 8 years

**DOI:** 10.1186/s40557-016-0132-1

**Published:** 2016-09-22

**Authors:** Sang Jin Park, Joo Hyun Sung, Chang Sun Sim, Seok Hyeon Yun, Jeong Han Yeom, Joong-Keun Kwon, Jiho Lee

**Affiliations:** 1 Department of Occupational and Environmental Medicine, Ulsan University Hospital, University of Ulsan College of Medicine, 877 Bangeojinsunhwando-ro, Dong-gu, Ulsan, 44033 Republic of Korea; 2 Department of Otorhinolaryngology, Ulsan University Hospital, University of Ulsan College of Medicine, 877, Bangeojinsunhwando-ro, Dong-gu, Ulsan, 44033 Republic of Korea

**Keywords:** Conductive hearing loss, Noise-induced hearing loss, Noise, Retrospective cohort study

## Abstract

**Background:**

The purpose of this study was to investigate hearing threshold changes of workers with unilateral conductive hearing loss who were exposed to workplace noise for 8-years.

**Methods:**

Among 1819 workers at a shipyard in Ulsan, 78 subjects with an air-bone gap ≥10 dBHL in unilateral ears were selected. Factors that could affect hearing were acquired from questionnaires, physical examinations, and biochemistry examinations. Paired *t*-test was conducted to compare the hearing threshold changes over time between conductive hearing loss (CHL) ear and sensorineural hearing loss (SNHL) ear.

**Results:**

The study included male subjects aged 48.7 ± 2.9, having worked for 29.8 ± 2.7 years. Hearing thresholds increased significantly in CHL ears and SNHL ears at all frequencies (0.5–6 kHz) during follow-up period (*p* < 0.05). The threshold change at 4 kHz was 3.2 dBHL higher in SNHL ears which was statistically significant (*p* < 0.05). When workers were exposed to noise levels of 85 dBA and above, threshold change at 4 kHz was 5.6 dBHL higher in SNHL ears which was statistically significant (*p <* 0.05). Among workers aged below 50, the threshold change values were lower in low-frequency (0.5–2 kHz) in SNHL ears, with a small range of changes, whereas in high-frequency (3–6 kHz), the range of changes was greater SNHL ears (*p* < 0.05). Among workers aged 50 and above, SNHL ears showed a wider range of changes in both high- and low-frequency areas (*p* < 0.05).

**Conclusions:**

At high-frequencies, particularly at 4 kHz, the range of hearing threshold changes was lower in ears with conductive hearing loss than in contralateral ears. This is suggested as a protective effect against noise exposure.

## Background

Hearing loss is one of the most frequent chronic sensorineural injuries. In 2013, The National Institute on Deafness and Other Communication Disorders reported the following statistics about hearing loss in adults: adults aged 45–54 showed a hearing loss level of 2 %, those aged 55–64 showed 8.5 %, 65–74 of 25 %, and 75 and above showed almost 50 % hearing loss [1]; 15 % of adults aged 20–69 showed high-frequency hearing loss induced by noise exposure [2].

According to “Workers’ periodic health examination”, a report produced by the Ministry of Employment and Labor in Korea in 2013, 25,891 workplaces had noisy working environment, and accordingly, 564,663 workers underwent hearing examination [2, 3]. South Korea regulates the continuous noise exposure limit at 90 dBA through the Industrial Safety and Health Act, a limit that is higher than the level of 85 dBA set by the National Institute for Occupational Safety and Health (NIOSH) [4]. Therefore, a significant proportion of workers may be exposed to hazardous noise levels.

An expected consequence of conductive hearing loss in these workers is a decrease in the degree of noise-related damage in the inner ears as a result of reduced sound energy transfer in the air conduction process. However, a few studies have investigated this. While a study on the protective effects of conductive hearing loss against excessive noise has been reported [4], the study has several limitations: the number of study subjects was small and the range of measured frequencies was limited. Furthermore, another study has reported that conductive hearing loss has no protective effect on the inner ears [5]. In summary, not many studies have investigated conductive hearing loss under noise exposure, and there is no clear conclusion among the studies that do exist.

In this study, we conducted an 8-year follow-up investigation on workers having unilateral conductive hearing loss (CHL), and compared the results with hearing changes in the contralateral ears. Until now, studies that observe long-term follow-up of conductive hearing loss have rarely been reported. Moreover, this study is the first of its kind to be conducted in South Korea. In addition to noise, this study analyzes the relation between hearing loss and other factors previously reported to affect hearing, including age [6, 7], smoking [8, 9], blood pressure [10, 11], diabetes mellitus [10, 12], and dyslipidemia [13, 14]. Our aim was to provide future directions for hearing management to workers with CHL.

## Methods

### Study population

**Fig. 1:**
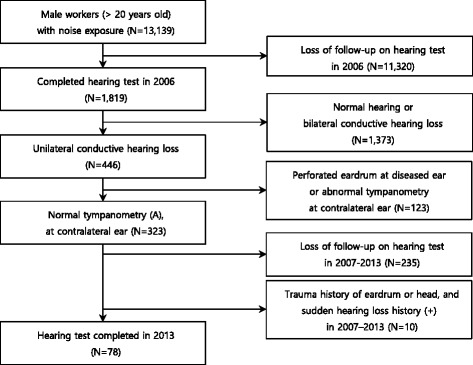
Flow chart of inclusion and exclusion criteria for subjects in 2006–2013

A total of 13,139 male workers aged 20–62 at a shipyard in Ulsan underwent special health examination for noise exposure, in 2006. Of these, 1819 workers completed audiometry examinations including bone conduction examination and tympanometry. These 1819 workers were selected as our initial subjects. There is controversy in definition of conductive hearing loss. Chung had defined as 15 dB or more air-bone gap for at least one frequency [15], on the other hand, Simpson et al., as 20 dB or greater averaged over 1, 2, and 3 kHz [5]. In this study, we defined cases in which an air-bone gap of 10 dBHL or higher appears in one or more frequencies (from among 0.5, 1, 2, 3, or 4 kHz) as conductive hearing loss. Out of a total of 446 subjects with unilateral conductive hearing loss, 78 subjects were finally selected. The remainder were excluded for the following reasons: cases of eardrum perforation in otoscopic examination, cases of atypical conductive hearing loss in the audiogram, cases of abnormal results from sensorineural hearing loss (SNHL) ear on tympanometry, cases of audiometry results omitted more than once during the study period, cases of trauma history of the eardrum or head in the past, and cases of sudden sensorineural hearing loss (Fig. 1).

### Questionnaire, physical examination and biochemistry

During the health examination procedures, subjects were asked to complete self-reported questionnaires. All information concerning age, working period, working department, history of ear disease, and trauma history of head was collected from these questionnaires. All damaged eardrum diagnoses were obtained from the otoscopy and tympanometry results. In order to investigate additional factors that may affect hearing, data regarding body mass index (BMI), blood pressure, fasting blood sugar (FBS), and total cholesterol were collected.

### Noise exposure level

Levels of noise in the work environment were measured in accordance with the Ministry of Employment and Labor Notification No. 2009-78 (dated February 14, 2009). Regarding noise-exposure levels, the subjects’ mean noise levels were calculated for each working department and process by using the regional and individual measurement results obtained in the first year of the research (2006). A noise dose badge (CR100, CIRRUS, England), audio dosimeter (MK-3, AMETEK, USA), two noise logging dosimeter (M-27 and 28, QUEST, USA) were used as the noise-level measuring devices, and the devices were adjusted with a sound calibrator before and after the use.

### Audiometry and tympanometry

Audiometry was conducted by experts trained through the quality assurance program for audiometry by the Korea Occupational Safety and Health Agency (KOSHA). The Hughson-Westlake procedure was used to detect air conduction thresholds at 0.5, 1, 2, 3, 4, and 6 kHz and to detect bone conduction thresholds at 0.5, 1, 2, 3, and 4 kHz. An AC40 diagnostic audiometer (Interacoustics, Denmark) with TDH-39P headphones attached was used for this procedure. An AT235 impedance audiometer (Interacoustics, Denmark) was used for tympanometry, and SDW-2000 (Interacoustics, Denmark) was used for soundproof booths. Audiometry and tympanometry were adjusted annually in accordance with the KOSHA guidelines, and the soundproof booths used were in accordance with American National Standards Institute (ANSI) S3.1-1999 [16].

### Statistical analysis

Air conduction threshold changes were investigated for the 8-year follow-up period (2006–2013) at each frequency for all SNHL and CHL ears. A paired *t*-test was conducted 1) to compare the differences between the air conduction threshold changes for the sensorineural hearing loss ears (*Δ*S) and those for the conductive hearing loss ears (*Δ*C), 2) to compare the hearing threshold changes between mean of low-frequency (0.5, 1, 2 kHz) and that of high-frequency (3, 4, 6 kHz).

All subjects were subsequently divided into two groups based on average work environment noise level (85 dBA) and age (50 and above). A paired *t*-test was used to compare mean of difference (2013-2006) between hearing threshold of SNHL ear and that of CHL ear, on air and bone conduction, according to average work environment noise level and age. SPSS 21.0 (IBM SPSS Incorporation, Chicago, IL, USA) was used for data analysis. When the *p*-value was lower than 0.05, we considered it statistically significant.

## Results

### General characteristics of subjects

The mean age of subjects at the start of the study was 48.7 ± 2.9 years (though 90 % subjects were aged 45 and above) and the mean length of their working period was 29.8 ± 2.7 years. BMI was 23.3 ± 1.8 kg/m^2^ and systolic blood pressure was 125.9 ± 12.5 mmHg. FBS and total cholesterol levels were 102.8 ± 17.7 mg/dL and 189.3 ± 31.2 mg/dL, respectively. Five subjects showed an FBS level above 126 mg/dL and seven subjects showed a total cholesterol level higher than 230 mg/dL. Among the 78 subjects, there were 14 smokers and 64 non-smokers. The mean noise level of their work environment was 83.5 ± 5.0 dBA, and 25 subjects were exposed to a noise level of 85 dBA or higher (Table 1).

**Table 1:** General characteristics of subjects at baseline (2006)

	Group (*N* = 78)	Distribution	Mean	SD^a^
Age (years)	<50 (40–49)	42 (53.85 %)	48.7	2.9
	≥50	36 (46.15 %)		
Working period (years)	20–29	43 (55.13 %)	29.8	2.7
	≥30	35 (44.87 %)		
Body mass index (BMI) (kg/m^2^)	<20.0	1 (1.28 %)	23.3	1.8
	20.0–22.9	28 (35.90 %)		
	≥ 23.0	49 (62.82 %)		
Systolic blood pressure (mmHg)	<120	17 (21.79 %)	125.9	12.5
	120–139	50 (64.10 %)		
	≥ 140	11 (14.10 %)		
Fasting blood sugar (mg/dL)	<100	37 (47.44 %)	102.8	17.7
	100–125	36 (46.15 %)		
	≥ 126	5 (6.41 %)		
Total cholesterol (mg/dL)	<200	52 (66.67 %)	189.3	31.2
	200–229	19 (24.36 %)		
	≥ 230	7 (8.97 %)		
Smoking status	Non-smoker	64 (82.05 %)	-	-
	Smoker	14 (17.95 %)		
Noise level (dBA)	< 85 (73.5–84.9)	53 (67.95 %)	83.5	5.0
	≥ 85	25 (32.05 %)		

^a^ *SD* standard deviation

### Comparison of hearing threshold changes in accordance with conductive hearing loss

**Fig. 2:**
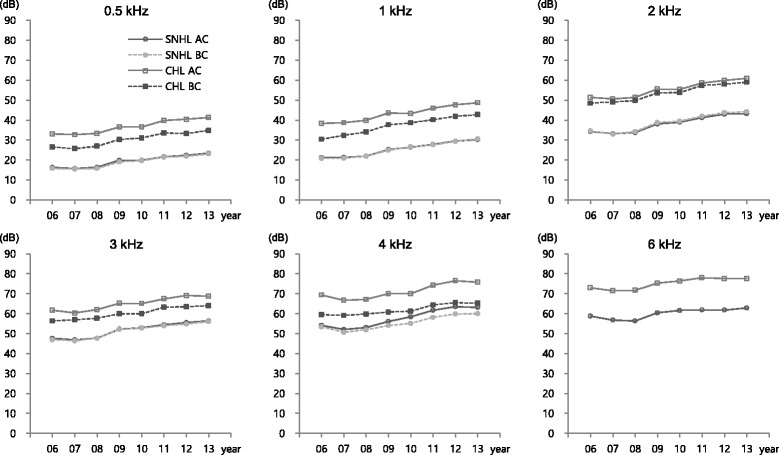
Hearing threshold changes for SNHL and CHL ear by frequency over 8 years (*N* = 78). SNHL AC, air conduction of sensorineural hearing loss ear; SNHL BC, bone conduction of sensorineural hearing loss ear; CHL AC, air conduction of conductive hearing loss ear; CHL BC, bone conduction of conductive hearing loss ear

The mean thresholds of air and bone conduction of SNHL and CHL ears increased over time at all frequencies during the study period. The reported values of the air-bone gap in the CHL ears at the initial stage of the study remained at a similar level during the follow-up period (Fig. 2).

In low-frequency areas, the observed hearing threshold changes were higher (8.7–11.2 dBHL) in CHL ears (compared to 7.9–10.3 dBHL in SNHL ears). In contrast, in high-frequency areas, hearing threshold changes were higher (4.7–9.4 dBHL) in SNHL ears (compared to 4.0–7.8 dBHL in CHL ears). On comparing hearing threshold changes between SNHL and CHL ears at each frequency, significantly 3.2 dBHL higher hearing threshold changes were detected at 4 kHz in SNHL ears than in CHL ears. However, significant differences were not detected at other frequencies (Table 2).

**Table 2:** Comparisons for hearing threshold changes of SNHL and CHL ear by frequency during follow-up period (*N* = 78)

	SNHL ear (*Δ*S)^a^ (dBHL)		CHL ear (*Δ*C)^b^ (dBHL)		Difference between *Δ*S and *Δ*C (dBHL)		
kHz	Mean	SD^c^	Mean	SD^c^	Mean	SD^c^	*p* ^*^
0.5	7.9	9.7	8.7	10.6	−0.8	8.0	0.389
1	10.3	10.1	11.2	10.7	−1.0	9.8	0.378
2	9.9	10.2	10.5	8.8	−0.6	9.6	0.604
3	9.4	10.2	7.8	9.4	1.6	9.8	0.164
4	8.9	8.5	5.6	9.3	3.2	10.2	0.006
6	4.7	12.0	4.0	10.6	0.7	13.2	0.638

^a^ *Δ*S, ^b^ *Δ*C, ^*^ *p*-value was calculated by paired *t*-test; ^a^ *Δ*S, difference of SNHL ear (2013-2006), *p* < 0.05; ^b^ *Δ*C, difference of CHL ear (2013-2006), *p* < 0.05; SD^c^, standard deviation

### Hearing threshold changes in accordance with noise-levels

**Fig. 3:**
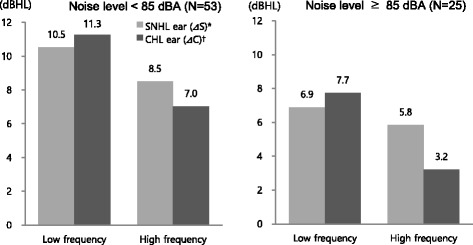
Comparisons of threshold changes between SNHL and CHL ear during follow-up period by noise level. Each value of columns was calculated by paired* t*-test.* p*<0.05; ^*^ *Δ*S=(average of air conduction threshold in 2013 at SNHL ear)–(average of air conduction threshold in 2006 at SNHL ear); ^†^ *Δ*C=(average of air conduction threshold in 2013 at CHL ear)–(average of air conduction threshold in 2006 at CHL ear)

The subjects were divided into groups according to mean noise levels experienced in the work environment (below 85 dBA or 85 dBA and above), and mean hearing threshold changes were investigated for each of the SNHL and CHL ears at each frequency. The range of changes was observed to be higher in CHL ears than in SNHL ears in low-frequency areas. Conversely, in high-frequency areas, the range was found to be higher in SNHL ears than in CHL ears. In subjects experiencing noise levels below 85 dBA, the mean hearing threshold ranges increased by 10.5 dBHL in SNHL ears and by 11.3 dBHL in CHL ears in the low-frequency areas. These ranges increased by 8.5 dBHL in SNHL ears and by 7 dBHL in CHL ears in the high-frequency areas (*p* < 0.05). In subjects experiencing noise levels of 85 dBA and above, the mean hearing threshold ranges increased by 6.9 dBHL in SNHL ears and by 7.7 dBHL in CHL ears in low-frequency areas, and increased by 5.8 dBHL in SNHL ears and by 3.2 dBHL in CHL ears in high-frequency areas (*p* < 0.05). Thus, hearing changes were more pronounced in low-frequency areas than in high-frequency areas (Fig. 3).

In subjects experiencing noise levels below 85 dBA, threshold changes of bone conduction at 0.5 kHz were observed to be 2.5 dBHL higher in CHL ears than in SNHL ears, which is statistically significant. In subjects experiencing noise levels of 85 dBA and above, threshold changes of air conduction at 4 kHz were observed to be 5.6 dBHL higher in SNHL ears than in CHL ears, which is again statistically significant (Table 3).

**Table 3:** Comparisons of threshold changes between SNHL and CHL ear during follow-up period by noise level

	Noise level < 85 dBA (*N* = 53)									Noise level ≥ 85 dBA (*N* = 25)								
	Difference of Air conduction			Difference of Bone conduction			Difference of Air-bone gap			Difference of Air conduction			Difference of Bone conduction			Difference of Air-bone gap		
kHz	Mean^a^	SD^d^	*p*	Mean^b^	SD^d^	*p*	Mean^c^	SD^d^	*p*	Mean^a^	SD^d^	*p*	Mean^b^	SD^d^	*p*	Mean^c^	SD^d^	*p*
0.5	−0.9	8.9	0.469	−2.5	8.2	0.027	1.7	7.8	0.125	−0.6	5.8	0.633	1.8	22.7	0.702	−2.3	23.0	0.618
1	−0.6	10.5	0.695	−2.1	10.4	0.147	1.5	8.1	0.175	−1.9	8.5	0.280	−3.5	8.6	0.051	1.6	4.8	0.098
2	−0.8	10.3	0.578	−0.5	11.0	0.757	−0.3	6.9	0.737	−0.1	8.0	0.960	−1.8	8.2	0.270	1.8	6.7	0.202
3	1.0	10.5	0.477	1.9	8.9	0.131	−0.8	7.6	0.432	2.7	8.3	0.118	0.6	9.9	0.750	2.0	7.8	0.202
4	2.2	10.7	0.149	0.3	7.3	0.794	1.9	7.9	0.087	5.6	8.8	0.004	2.1	6.6	0.124	3.4	6.7	0.017
6	1.2	13.0	0.503	-	-	-	-	-	-	−0.4	13.7	0.896	-	-	-	-	-	-

*p*-value was calculated by paired *t*-test; ^a^Mean, difference of air conduction threshold (2013-2006) at SNHL ear vs. difference of air conduction threshold (2013-2006) at CHL ear; ^b^Mean, difference of bone conduction threshold (2013-2006) at SNHL ear vs. difference of bone conduction threshold (2013-2006) at CHL ear; ^c^Mean, average of air-bone gap; ^d^ *SD* standard deviation

### Hearing threshold changes in accordance with age

**Fig. 4:**
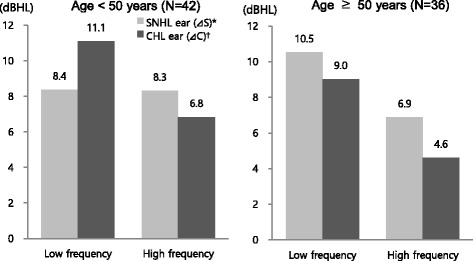
Comparisons of threshold changes between SNHL and CHL ear during follow-up period by age. Each value of columns was calculated by paired* t*-test.* p*<0.05; ^*^ *Δ*S=(average of air conduction threshold in 2013 at SNHL ear)–(average of air conduction threshold in 2006 at SNHL ear); ^†^ *Δ*C=(average of air conduction threshold in 2013 at CHL ear)–(average of air conduction threshold in 2006 at CHL ear)

The subjects were divided into groups by age, and hearing threshold changes were analyzed. The thresholds of air and bone conduction were increased in SNHL and CHL ears in 2013 (in comparison with 2006). In subjects aged below 50, the mean hearing threshold ranges increased by 8.4 dBHL in SNHL ears and by 11.1 dBHL in CHL ears in low-frequency areas (*p* < 0.05), whereas the ranges increased by 8.3 dBHL in SNHL ears and by 6.8 dBHL in CHL ears in high-frequency areas (*p* < 0.05). In subjects aged 50 and above, the mean hearing threshold ranges increased by 10.5 dBHL in SNHL ears and by 9.0 dBHL in CHL ears in low-frequency areas (*p* < 0.05), whereas the ranges increased by 6.9 dBHL in SNHL ears and by 4.6 dBHL in CHL ears in high-frequency areas (*p* < 0.05). Hearing threshold changes were observed to be higher in low-frequency areas than in high-frequency areas in both age groups. However, in subjects aged 50 and above, the range was higher in SNHL ears than in CHL ears in both low- and high-frequency areas. In contrast, in subjects aged below 50, the range of changes was observed to be higher in CHL ears than in SNHL ears in low-frequency areas, and it was higher in SNHL ears than in CHL ears in high-frequency areas (Fig. 4).

The hearing threshold changes were calculated for paired SNHL and CHL ears at each frequency and were then compared directly. In subjects aged below 50, the threshold of air conduction at 1 kHz was observed to be significantly lower (by 3.7 dBHL) in SNHL ears than in CHL ears, and the threshold of bone conduction was observed to be significantly lower (by 4.0 dBHL) in SNHL ears than in CHL ears. In subjects aged 50 and above, the threshold changes of air conduction in SNHL ears at 4 kHz was observed to be significantly higher (by 4.1 dBHL) than that in CHL ears (Table 4).

**Table 4:** Comparisons of threshold changes between SNHL and CHL ear during follow-up period by age

	Age < 50 years (*N* = 42)									Age ≥ 50 years (*N* = 36)								
	Difference of Air conduction			Difference of Bone conduction			Difference of Air-bone gap			Difference of Air conduction			Difference of Bone conduction			Difference of Air-bone gap		
kHz	Mean^a^	SD^d^	*p*	Mean^b^	SD^d^	*p*	Mean^c^	SD^d^	*p*	Mean^a^	SD^d^	*p*	Mean^b^	SD^d^	*p*	Mean^c^	SD^d^	*p*
0.5	−2.0	8.7	0.136	−1.9	8.6	0.158	−0.1	5.9	0.875	0.7	6.8	0.544	−0.3	19.4	0.925	1.0	20.4	0.771
1	−3.7	9.5	0.017	−4.0	9.6	0.011	0.3	5.3	0.729	2.1	9.4	0.180	−0.9	9.9	0.582	3.1	8.7	0.042
2	−2.5	9.8	0.110	−2.3	9.1	0.113	−0.2	6.7	0.837	1.7	8.9	0.268	0.7	11.2	0.724	1.0	7.1	0.404
3	1.4	11.2	0.420	1.9	9.7	0.220	−0.5	8.6	0.735	1.8	8.2	0.208	1.0	8.7	0.481	0.7	6.6	0.518
4	2.5	10.8	0.134	1.3	7.3	0.271	1.3	7.7	0.283	4.1	9.5	0.015	0.4	7.0	0.740	3.7	7.3	0.005
6	0.5	15.0	0.838	-	-	-	-	-	-	1.0	10.8	0.593	-	-	-	-	-	-

*p*-value was calculated by paired *t*-test; ^a^Mean, difference of air conduction threshold (2013-2006) at SNHL ear vs. difference of air conduction threshold (2013-2006) at CHL ear; ^b^Mean, difference of bone conduction threshold (2013-2006) at SNHL ear vs. difference of bone conduction threshold (2013-2006) at CHL ear; ^c^Mean, average of air-bone gap; ^d^SD, standard deviation

## Discussion

Hearing loss is characterized by a decreased state of sensitivity toward a specific sound. By decreasing cognitive function towards externally induced sounds and signals, hearing loss can crucially impair an individual’s reaction to dangerous stimuli. Auditory sensation is a fundamental human sensation and plays a very important part in maintaining one’s quality of life. However, many workers in South Korea are frequently exposed to hazardous noise at all times, and are constantly in danger from noise-induced hearing loss (NIHL). Workplaces where the noise level at the work environment exceeds the permissible level (or where NIHL has previously occurred) are placed under constant supervision, and the employers are advised to implement a hearing-conservation program. However, because of the unwelcome economic burden of noise reduction placed on employers and the lukewarm responses towards these measures shown by workers themselves, NIHL comprises the majority of cases of occupational diseases reported since the 1990s, and this trend is expected to continue.

In this study, the hearing ability of workers with unilateral conductive hearing loss was analyzed, and the results compared with those of contralateral ears to investigate how existing conductive hearing loss affects the progress of NIHL. Hearing has been reported to deteriorate with age [7]. During the follow-up period, air conduction threshold and bone conduction threshold increased significantly in both SNHL and CHL ears, indicating the deterioration of hearing over time. These results represent the potential effects of aging or noise exposure while working.

Regarding hearing threshold changes of air and bone conduction at each frequency, the air-bone gaps were not observed in SNHL ears for most frequencies during the follow-up period; however, in CHL ears, the air-bone gaps observed in the early period of study, and persisted during the follow-up period, without much change. This indicates the air-bone gap induced by conductive hearing loss consistently affects the subjects’ hearing threshold level without any improvement over time. In calculating the changes in SNHL and CHL ears at each frequency and comparing the differences, the results showed that the changes in SNHL ears were significantly greater than the changes in CHL ears (by 3.2 dBHL at 4 kHz). Thus, when conductive hearing loss is a precondition, hearing deterioration induced by noise exposure is comparatively small.

In the two noise-level groups (below 85 dBA vs. 85 dBA and above), hearing ability was observed to gradually decrease over time in both CHL and SNHL ears. However, when considering the differences in threshold changes between SNHL and CHL ears at each frequency, significant differences were observed in threshold changes of bone conduction at 0.5 kHz, at noise levels below 85 dBA. At noise levels of 85 dBA and above, as shown in the previous result, threshold changes of air conduction were significantly greater at 4 kHz in SNHL ears than in CHL ears. These results indicate that, at 4 kHz, the threshold changes of air conduction were smaller in CHL ears than in SNHL ears. In other words, conductive hearing loss is associated with a decrease in noise-induced injuries. Our interpretation of this result is that, in agreement with Chung’s study [15], CHL ears (with conductive hearing loss) are less affected by noise exposure.

When looking at hearing threshold changes in subjects categorized in accordance with age (below 50 vs. 50 and above), hearing deteriorated in both ears in the two groups over time. When comparing difference of the changes in SNHL and CHL ears at each frequency, the air and bone conduction threshold changes at 1 kHz were significantly greater in CHL ears than in SNHL ears in subjects aged below 50. This result indicates that in relatively younger subjects, hearing deterioration induced by conductive hearing loss continues in low-frequency over time. On the other hand, in the subjects aged 50 and above, significant differences were not detected at 1 kHz, and threshold changes of air conduction in SNHL ears at 4 kHz were significantly greater than that in CHL ears. This result suggested that in relatively older subjects, hearing deterioration induced by noise exposure can be accelerated in high-frequency over time. In other words, hearing in the ears without conductive hearing loss gets affected by noise exposure to a greater extent in the younger age group and to a smaller extent in the higher age group. The reasons for this can be found in aging-induced overall hearing damage. In particular, in cases involving relatively older subjects, high-frequency hearing loss were observed in the early stage of the study, thus, this ceiling effect might have caused the relatively lower changes observed during the follow-up period. Gates et al. investigated the correlation between age and noise-induced injury with 203 adults included in the Framingham Heart Study cohort (aged 58–80) as the subjects. The subjects were categorized into three groups according to the audiogram results obtained in the early stage of the study. The threshold increasing patterns in high-frequency (i.e., notch) were then evaluated, and the changes in the patterns were confirmed after the follow-up period [16]. The result showed that as age increase, the rate of hearing loss in low-frequency (especially at 2 kHz) accelerates whereas the rate of hearing loss in high-frequency (3, 4, and 6 kHz) slows down. The results of this study also showed that low-frequency hearing loss in older subjects was greater in SNHL ears than in CHL ears. Thus, we assume that, in agreement with the above results, hearing deterioration accelerated in low-frequency because of aging.

The C5-dip is observed in NIHL demonstrates a remarkable increase of the hearing threshold in the audiogram at 4 kHz compared to other frequencies. This study shows that hearing threshold changes were observed to be significant greater in SNHL ears than in CHL ears at around 4 kHz, and in accordance with noise levels, these differences continued only when exposed to noise levels of 85 dBA and above. This indicates that hearing threshold changes are greater in SNHL ears than in CHL ears at 4 kHz, that is, CHL ears are affected lesser by noise exposure than SNHL ears are. Thus, it can be concluded that a protective effect against noise exists in CHL ears in comparison with in SNHL ears.

In cases involving middle ear diseases, such as chronic otitis media, a decrease in eardrum elasticity or an eardrum perforation, accompanied in part by loss of the ossicles, there is a decrease in sound energy transfer through the eardrum and ossicles to the inner ear. The cochlea resonates at frequency-specific locations, that is high-frequency sounds with short wavelengths are received at the base and low-frequency sounds with long wavelengths are received near to the apex. The precise mechanism of occurrence of NIHL is unknown. Pathologic lesions have been observed in the outer and inner hair cells of the inner ear in previous studies on humans and animals, and our understanding of the occurrence of noise-induced deformation of inner ear organs is based on these findings [17–22]. Noise-induced hair cell damages become predominant in high-frequency receiving areas. In cases with conductive hearing loss, a decrease in the ability to receive sound energy is expected in the high-frequency receiving areas close to the middle ear. As a result, the energy transfer of high-frequency sound decreases, thereby reducing hair cell damage at obnoxious noise levels, and this results in a protective effect against noise.

The effects of reported influencing variables on hearing threshold changes were analyzed by multiple linear regression tests, although it is not shown in the table. Except for CHL ears at 2 kHz, a significant positive association with FBS was detected in low-frequency areas. Systolic blood pressure showed significant negative association in SNHL ears at 1 kHz. In terms of the noise level, a significant negative association was detected in SNHL ears at 0.5 and 1 kHz, also at 3 and 4 kHz in CHL ears.

The strengths of this study are as follows: This study conducted an 8-year follow-up for the first time in South Korea on noise exposure effects with workers who have unilateral conductive hearing loss as the subjects in comparison with hearing changes in contralateral ears. Previous studies that have investigated a long-term follow-up in this topic are rare overseas as well. Furthermore, hearing thresholds of individuals on both sides were compared and analyzed, minimizing the effects induced by individual characteristics. Several previous studies have compared hearing thresholds of individuals on both sides on identical individuals as subjects, but the measured frequency ranges have been limited. Moreover, they have been mostly cross-sectional studies, thereby limited in confirming causal relationships [5, 15].

The limitations of this study are as follows: First, the subjects were selected using initial audiometry results because the confirmation of the onset of conductive hearing loss is impossible. Second, most of the subjects (over 90 %) were aged 45 and above, with over 46 % at 50 and above, and thus, age-induced hearing loss could not be excluded. Third, the subjects’ working periods were mostly over 25 years, and thus in most cases susceptibility to noise is assumed to have decreased. Moreover, there was no noticeable tendency for the susceptibility to change during the study period. The protective effect of conductive hearing loss under noise exposure was observed at some frequencies even though the evidence was not conclusive. In the future, additional follow-up studies are required to examine the early effects of noise exposure on relatively young male and female workers with working periods of less than 20 years, or to observe changes in workers’ hearing level to noise exposure immediately after their otologic surgery.

## Conclusions

During the 8-year follow-up period, this study investigated 78 workers with unilateral conductive hearing loss who have been exposed to workplace noise. Hearing threshold changes in the ears with conductive hearing loss was significantly smaller than that in the contralateral ears at 4 kHz, though statistically significant differences were not found in other frequency area. From the results of this study, we suggest that protective effects are against noise in workers with CHL. Further studies are needed to investigate early effects of noise exposure on relatively young male and female workers with working periods of less than 5 years to complement this study.

## Abbreviations

CHL: Conductive hearing loss
SNHL: Sensorineural hearing loss
NIHL: Noise-induced hearing loss
NIOSH: National Institute for Occupational Safety and Health
BMI: Body mass index
FBS: Fasting blood sugar
KOSHA: Korea Occupational Safety and Health Agency
